# CTX-M-14 and CTX-M-15 enzymes are the dominant type of extended-spectrum *β*-lactamase in clinical isolates of *Escherichia coli* from Korea

**DOI:** 10.1099/jmm.0.004507-0

**Published:** 2009-02

**Authors:** Wonkeun Song, Hyukmin Lee, Kyungwon Lee, Seok Hoon Jeong, Il Kwon Bae, Jae-Seok Kim, Hyo-Sun Kwak

**Affiliations:** 1 Department of Laboratory Medicine, Hallym University College of Medicine, 150-950, 948-1 Daerim 1-Dong, Youngdeungpo-Gu, Seoul, Republic of Korea; 2 Department of Laboratory Medicine, Kwandong University College of Medicine, 697-24 Whajung-Dong, Dukyang-Gu, Goyang, Republic of Korea; 3 Department of Laboratory Medicine and Research Institute of Bacterial Resistance, Yonsei University College of Medicine, 120-752, 134 Shinchon-Dong, Seodaemu-Gu, Seoul, Republic of Korea; 4 Research Institute for Antimicrobial Resistance, Kosin University College of Medicine, 602-030, 34 Amnam-Dong, Suh-Gu, Busan, Republic of Korea; 5 Department of Laboratory Medicine, Hallym University College of Medicine, 134-701, 150 Sungnae-Gil, Kangdong-Gu, Seoul, Republic of Korea; 6 Center for Food Safety Evaluation, Korea Food and Drug Administration, 122-704, 231 Jinheung-Ro, Eunpyung-Gu, Seoul, Republic of Korea

## Abstract

This study was performed to assess the prevalence and genotypes of plasmid-borne extended-spectrum *β*-lactamases (ESBLs) and AmpC *β*-lactamases in *Escherichia coli* in Korea. A total of 576 isolates of *E. coli* was collected from 12 Korean hospitals during May and July 2007. A phenotypic confirmatory test detected ESBLs in 82 (14.2 %) of the 576 *E. coli* isolates. The most common types of ESBLs identified were CTX-M-14 (*n*=32) and CTX-M-15 (*n*=27). The prevalence and diversity of the CTX-M mutants, including CTX-M-15, CTX-M-27 and CTX-M-57, with significant hydrolytic activity against ceftazidime were increased. PCR experiments detected genes encoding plasmid-borne AmpC *β*-lactamases in 15/56 cefoxitin-intermediate or cefoxitin-resistant isolates, and the most common type of AmpC *β*-lactamase identified was DHA-1 (*n*=10). These data suggest that the incidence of ESBLs in *E. coli* has increased as a result of the dissemination of CTX-M enzymes in Korea. In addition, CTX-M-22, CTX-M-27 and CTX-M-57 have appeared in Korea.

## INTRODUCTION

The predominant mechanism for acquired resistance to *β*-lactams in *Escherichia coli* is the synthesis of plasmid-borne extended-spectrum *β*-lactamases (ESBLs) and AmpC *β*-lactamases. The clavulanic acid (CA)-inhibitory ESBLs are within molecular class A of the Ambler classification scheme and confer resistance to oxyimino-cephalosporins on their bacterial hosts (Paterson & Bonomo, 2005). CTX-M enzymes are spreading rapidly and are now the dominant type of ESBL in *E. coli* in many parts of the world (Rossolini *et al.*, 2008), although classical TEM and SHV ESBLs are still dominant in the USA (Bush, 2008). In Korea, members of the CTX-M-1 and CTX-M-9 clusters have repeatedly been reported in *Enterobacteriaceae* since the first finding of CTX-M-14 in clinical isolates of *Shigella sonnei*, *E. coli* and *Klebsiella pneumoniae* in 2001 (Pai *et al.*, 2001). A nationwide survey in 2003 reported 23/246 clinical isolates (9.3 %) of *E. coli* with an ESBL phenotype, and only 8 of these had CTX-M enzymes (Ryoo *et al.*, 2005).

*E. coli* is intrinsically susceptible to 7-*α*-methoxy-cephalosporins [e.g. cefoxitin (FOX) and cefotetan] because of the low-level expression of the non-inducible species-specific *ampC* gene (Philippon *et al.*, 2002). In 1989, Bauernfeind *et al.* (1989) described a FOX-resistant *K. pneumoniae* isolate, which produced a plasmid-borne AmpC *β*-lactamase, named CMY-1, from Korea. Subsequently, various types of plasmid-borne AmpC enzymes have been found worldwide (Philippon *et al.*, 2002). In Korea, FOX-resistant *E. coli* have increasingly been noted, and a study performed in 2003 showed that a high portion (53.4 %, 62/116) of FOX resistance in *E. coli* was due to plasmid-borne AmpC *β*-lactamase production (Lee *et al.*, 2006). The aim of the present study was to determine the prevalence and shift of plasmid-borne AmpC *β*-lactamases and ESBLs, with a special focus on the CTX-M enzymes, in *E. coli* in Korea.

## METHODS

### Bacterial strains.

Consecutive non-duplicate isolates of *E. coli* were collected during May and July 2007 from 12 hospitals in nine cities in Korea. The isolates were identified using the API-20 E system (bioMérieux). The azide-resistant *E. coli* J53 was used as a recipient strain for conjugative transfer. *E. coli* ATCC 25922 and *K. pneumoniae* ATCC 700603 were used as MIC reference strains.

### Antimicrobial susceptibility testing.

Antibiotic-containing discs (BBL) were used for routine antibiograms by disc diffusion assay (CLSI, 2006a). The phenotypic confirmatory test for ESBL and/or AmpC *β*-lactamase production using boronic acid (BA) as an AmpC *β*-lactamase inhibitor was performed as described previously (Song *et al.*, 2007). MICs were determined by the agar dilution method using Mueller–Hinton agar (MHA; Difco Laboratories) with an inoculum of 10^4^ c.f.u. (CLSI, 2006b). The MICs of *β*-lactams were determined alone or in combination with a fixed concentration (4 μg ml^−1^) of CA.

### Mating-out assays.

Conjugation experiments were carried out between donors and the azide-resistant recipient strain *E. coli* J53 on MHA plates. Transconjugants were selected on MHA plates supplemented with 2 μg ceftazidime (CAZ) ml^−1^ or 2 μg cefotaxime (CTX) ml^−1^ and 100 μg sodium azide ml^−1^.

### Characterization of genes encoding *β*-lactamases.

Detection of genes encoding plasmid-borne ESBLs and AmpC *β*-lactamases was performed by PCR amplification with the primers listed in Table 1, as described previously (Ryoo *et al.*, 2005; Song *et al.*, 2006). Templates for PCR amplification in the clinical isolates were plasmid preparations from the clinical isolates. The PCR products were subjected to direct sequencing. Both strands of all PCR products were sequenced twice using an automatic sequencer (model 3730xl; Applied Biosystems).

## RESULTS

### *E. coli* isolates harbouring ESBLs

In the study period, clinical *E. coli* isolates (*n*=576) were obtained from outpatients (46.2 %), inpatients of general wards (46.9 %) and inpatients of intensive care units (6.9 %). These isolates were obtained from urine (70.3 %), blood (8.3 %), wound (7.4 %), sputum (6.2 %) and other samples (7.8 %). The phenotypic confirmatory test with BA detected ESBLs in 82 (14.2 %) *E. coli* isolates. Clinical isolates with an ESBL phenotype were found in all 12 hospitals (Table 2). Transfer of CAZ or CTX resistance determinants to the azide-resistant *E. coli* J53 recipient by conjugation was successful in 56/82 isolates with an ESBL phenotype.

PCR experiments detected genes encoding members of the CTX-M-1 and CTX-M-9 clusters in 42 and 35 isolates, respectively. The most common types of class A ESBLs identified were CTX-M-14 (*n*=32) and CTX-M-15 (*n*=27). Genes encoding CTX-M-3 (*n*=10), CTX-M-9 (*n*=2), CTX-M-12 (*n*=1), CTX-M-22 (*n*=2), CTX-M-27 (*n*=1) and CTX-M-57 (*n*=2) were also detected. The *bla* gene of SHV-12 was detected in two isolates. Genes encoding TEM-type *β*-lactamases were detected in 62/82 isolates, but all were TEM-1. Non-TEM and non-SHV ESBLs, including PER, VEB, GES and TLA enzymes and members of the CTX-M-2 and CTX-M-8 clusters, were not detected in this survey. No ESBL was detected in 4/82 isolates with an ESBL phenotype. These isolates may have another ESBL-encoding gene not detected with our primers or may have had false-positive results for ESBL activity.

### *E. coli* isolates harbouring AmpC *β*-lactamases

Among 56 FOX-intermediate or FOX-resistant isolates, 26 exhibited positive results in the phenotypic confirmatory test, i.e. a ≥5 mm increase in the zone diameter of either the FOX or the cefotetan disc in the presence of BA. PCR experiments detected genes encoding plasmid-borne AmpC *β*-lactamases in 15 isolates. The most common type of AmpC *β*-lactamase identified was DHA-1 (*n*=10), and genes encoding CMY-2 (*n*=3), CMY-10 (*n*=1) and CMY-11 (*n*=1) were also detected. Nine of the fifteen isolates simultaneously harboured ESBLs.

### Phenotypic characteristics

All of the isolates producing CTX-M-3 (*n*=10), CTX-M-9 (*n*=1), CTX-M-14 (*n*=30) or CTX-M-22 (*n*=1) had more than eightfold higher MICs for CTX than for CAZ (Table 3). The isolates producing CTX-M-15, CTX-M-27 or CTX-M-57 exhibited a high level of resistance to CAZ. Fourteen isolates producing plasmid-borne AmpC *β*-lactamases were highly resistant to FOX (MICs ≥128 mg l^−1^), except for two producing DHA-1. All of the isolates producing ESBLs exhibited a high level of resistance to ciprofloxacin (MICs ≥64 mg l^−1^), except for three (data not shown). A total of 15 out of 82 isolates (18.3 %) with an ESBL phenotype were resistant to amikacin (MICs ≥32 mg l^−1^).

## DISCUSSION

Compared with a survey in 1997 (Pai *et al.*, 1999), the prevalence of ESBL-producing *E. coli* in Korea has increased threefold from 4.8 to 14.2 %. We have shown previously that only 3.3 % (8/246) of clinical *E. coli* isolates produced CTX-M ESBLs in 2003 (Ryoo *et al.*, 2005), but the prevalence of these enzymes increased to 13.4 % (77/576) in the present study. These results indicate that the significant increase in ESBL incidence in *E. coli* may be due to dissemination of CTX-M enzymes. The incidence of CTX-M-14 and CTX-M-15 increased from 0.4 % (1/246) and 1.6 % (4/246) to 5.6 % (32/576) and 4.7 % (27/576), respectively, during this period (Ryoo *et al.*, 2005).

Dissemination of *E. coli* harbouring members of the CTX-M-1 and CTX-M-9 clusters has repeatedly been reported in Asian countries (Hawkey, 2008). CTX-M-14 was the dominant type of ESBL in *E. coli* from China (223/325) and Taiwan (88/128) (Yan *et al.*, 2006; Yu *et al.*, 2007), and CTX-M-3 was the second most frequent type in these countries (53/325 and 33/128, respectively). Interestingly, CTX-M-15 was rarely detected in these countries (2/325 and 2/128, respectively), whilst the enzyme was the second most dominant type of ESBL in Korea. Three CTX-M enzymes, including CTX-M-22, CTX-M-27 and CTX-M-57, were detected for what is believed to be the first time in Korea in the present study.

It is worth noting that the prevalence and diversity of the CTX-M mutants, including CTX-M-15, CTX-M-27 and CTX-M-57, with significant hydrolytic activity against CAZ have increased (Poirel *et al.*, 2002; Bonnet *et al.*, 2003; Hopkins *et al.*, 2008). Past reports have shown that the most common ESBL in *E. coli* isolates from Korea was TEM-52 (Pai *et al.*, 1999), but this ESBL was not detected in our study. Furthermore, SHV-12, which was the most common ESBL in *K. pneumoniae* in 2003 (Ryoo *et al.*, 2005), was detected in only two isolates in this study. Thus, it appears that CTX-M enzymes with an expanded activity towards CAZ in *E. coli* may be replacing TEM-52 and SHV-12, which confers a high level of resistance against CAZ on their bacterial hosts.

A survey in 2003 showed that the most common plasmid-borne AmpC *β*-lactamase in *E. coli* isolates from Korea was CMY-2 (Lee *et al.*, 2006), but it was detected in only three isolates in this study. The present study showed that the most common AmpC *β*-lactamase in *E. coli* isolates was DHA-1, an inducible enzyme, and the proportion of AmpC *β*-lactamase-producing isolates among the ESBL-producing and -non-producing isolates was significantly different: 11.0 % (9/82) versus 1.2 % (6/494), respectively.

The present data suggest that the incidence of isolation of ESBLs in *E. coli* has increased as a result of the dissemination of CTX-M enzymes in Korea. The most common ESBL and AmpC *β*-lactamase in *E. coli* were CTX-M-14 and DHA-1, respectively. In addition, CTX-M-22, CTX-M-27 and CTX-M-57 have appeared in Korea.

## Figures and Tables

**Table 1. t1:** Primers used in this study

**PCR target**	**Primer name**	**Primer sequence (5′→3′)**
*bla*_CTX-M_ (CTX-M-1 cluster)	CTX-M-1F	CCGTCACGCTGTTGTTAGG
	CTX-M-1R	GACGATTTTAGCCGCCGAC
	BM-1F	ACTATGGCACCACCAACGAT
	FM-1R	TTCGGTTCGCTTTCACTTTT
*bla*_CTX-M_ (CTX-M-2 cluster)	CTX-M-2F	CGGTGCTTAAACAGAGCGAG
	CTX-M-2R	CCATGAATAAGCAGCTGATTGCCC
*bla*_CTX-M_ (CTX-M-8 cluster)	CTX-M-8F	ACGCTCAACACCGCGATC
	CTX-M-8R	CGTGGGTTCTCGGGGATAA
*bla*_CTX-M_ (CTX-M-9 cluster)	CTX-M-9F	GATTGACCGTATTGGGAGTTT
	CTX-M-9R	CGGCTGGGTAAAATAGGTCA
*bla*_TEM_	TEM-F	ATGAGTATTCAACATTTCCGT
	TEM-R	TTACCAATGCTTAATCAGTGA
*bla*_SHV_	SHV-F	CCGGGTTATTCTTATTTGTCGCT
	SHV-R	TAGCGTTGCCAGTGCTCG
*bla*_PER-1_	PER-1F	GTTAATTTGGGCTTAGGGCAG
	PER-1R	CAGCGCAATCCCCACTGT
*bla*_VEB_	VEB-F	ACCAGATAGGAGTACAGACATATGA
	VEB-R	TTCATCACCGCGATAAAGCAC
*bla*_GES/IBC_	GES/IBC-F	GTTAGACGGGCGTACAAAGATAAT
	GES/IBC-R	TGTCCGTGCTCAGGATGAGT
*bla*_TLA_	TLA-F	CGCGAAAATTCTGAAATGAC
	TLA-R	AGGAAATTGTACCGAGACCCT
*bla*_DHA-1_	DHA-F	GGGGAGATAACGTCTGACCA
	DHA-R	TAGCCAGATCCAGCAATGTG
*bla*_CMY-1_	CMY-1F	TCACATCGGCTTCACAGAGC
	CMY-1R	CCATGGTGATGCTGTCAAAGA
*bla*_CMY-2_	CMY-2F	CAACACGGTGCAAATCAAAC
	CMY-2R	CATGGGATTTTCCTTGCTGT
*bla*_ACT-1_	ACT-1F	CGTCATGGTCTCGTCCGTTAG
	ACT-1R	CCTTGACCTCATCCGGTACCT

**Table 2. t2:** Distribution of ESBL and AmpC *β*-lactamases in *E. coli* isolates from Korea

**Type of *β*-lactamase**	No. of isolates collected from each hospital*												**Total no. of isolates (*n*=576)**
	**SL1 (*n*=40)**	**SL2 (*n*=49)**	**SL3 (*n*=50)**	**SL4 (*n*=50)**	**GY (*n*=50)**	**SN (*n*=50)**	**SW (*n*=50)**	**WJ (*n*=50)**	**GM (*n*=49)**	**BS (*n*=50)**	**GJ (*n*=50)**	**JJ (*n*=38)**	
CTX-M-1 cluster	5	3	2	3	1	3	3	5	4	5	5	3	42
CTX-M-3			1	1		1		3	2	1	1		10
CTX-M-12+DHA-1											1		1
CTX-M-15	5	2	1		1	2	2	2	2	2	2	3	24
CTX-M-15+DHA-1				1									1
CTX-M-15+CMY-2											1		1
CTX-M-15+CMY-10							1						1
CTX-M-22										1			1
CTX-M-22+DHA-1										1			1
CTX-M-57		1											1
CTX-M-57+DHA-1				1									1
CTX-M-9 cluster	2	4	4	2	1	0	6	8	2	3	1	2	35
CTX-M-9										1			1
CTX-M-9+DHA-1										1			1
CTX-M-14	2	2	4	1	1		6	8	2	1	1	2	30
CTX-M-14+SHV-12		1											1
CTX-M-14+CMY-2				1									1
CTX-M-27		1											1
SHV-12+DHA-1							1						1
DHA-1		3	1										4
CMY-2			1										1
CMY-11											1		1
Unidentified†				1	1					2			4
Total	7	10	8	6	3	3	10	13	6	10	7	5	88

*The 12 hospitals were in Seoul (SL1–4), Goyang (GY), Sungnam (SN), Suwon (SW), Wonju (WJ), Gumi (GM), Busan (BS), Gwangju (GJ) and Jeju (JJ) in Korea. †The BA disc test for ESBLs was positive, but no ESBL or *ampC* genes were detected.

**Table 3. t3:** MICs (μg ml^−1^) for 82 *E. coli* isolates with ESBL and/or AmpC enzymes

**Type of *β*-lactamase (no. of isolates)**	**FOX**			**CAZ**			CAZ–CA*			**CTX**			CTX–CA*		
	**Range**	**MIC_50_**	**MIC_90_**	**Range**	**MIC_50_**	**MIC_90_**	**Range**	**MIC_50_**	**MIC_90_**	**Range**	**MIC_50_**	**MIC_90_**	**Range**	**MIC_50_**	**MIC_90_**
CTX-M-3 (10)	4–32	8	32	2–32	4	32	0.3–1	1	1	16–>256	128	>256	0.1–1	0.3	1
CTX-M-12+DHA-1 (1)	256			256			128			64			32		
CTX-M-15 (24)	4–64	16	32	16–>256	64	>256	0.3–4	1	4	128–>256	>256	>256	0.1–1	0.5	1
CTX-M-15+DHA-1 (1)	128			64			2			>256			1		
CTX-M-15+CMY-2 (1)	256			64			64			>256			32		
CTX-M-15+CMY-10 (1)	>256			64			16			256			128		
CTX-M-22 (1)	2			1			0.1			32			0.1		
CTX-M-22+DHA-1 (1)	256			32			2			32			1		
CTX-M-57 (1)	64			32			1			256			0.3		
CTX-M-57+DHA-1 (1)	256			64			8			256			2		
CTX-M-9 (1)	4			1			0.3			32			0.1		
CTX-M-9+DHA-1 (1)	128			2			1			>256			0.5		
CTX-M-14 (30)	2–128	16	64	0.3–16	2	8	0.1–2	1	2	16–>256	128	256	0.1–1	0.5	1
CTX-M-14+SHV-12 (1)	16			256			1			64			0.1		
CTX-M-14+CMY-2 (1)	256			64			64			128			32		
CTX-M-27 (1)	16			256			1			>256			0.3		
SHV-12+DHA-1 (1)	>256			64			8			16			4		
DHA-1 (4)	16–256			0.1–32			0.1–8			0.1–32			0.1–8		
CMY-2 (1)	256			16			16			16			32		
CMY-11 (1)	>256			16			16			128			128		

*CA at a fixed concentration of 4 μg ml^−1^.

## Abbreviations

- BA, boronic acid
- CA, clavulanic acid
- CAZ, ceftazidime
- CTX, cefotaxime
- ESBL, extended-spectrum *β*-lactamase
- FOX, cefoxitin
